# Characterization of β-lactam resistance in *K*. *pneumoniae* associated with ready-to-eat processed meat in Egypt

**DOI:** 10.1371/journal.pone.0238747

**Published:** 2020-09-03

**Authors:** Shaymaa H. Abdel-Rhman

**Affiliations:** Microbiology and Immunology Department, Faculty of Pharmacy, Mansoura University, Mansoura, Egypt; Panstwowy Instytut Weterynaryjny - Panstwowy Instytut Badawczy w Pulawach, POLAND

## Abstract

*K*. *pneumoniae* was known as a nosocomial infection that causes human diseases. It is considered as one of the food-borne pathogens as it causes septicemia and diarrhea in humans. This study aims to characterize *K*. *pneumoniae* strains isolated from ready to eat processed meat phenotypically and genetically. Three hundred and fifty ready to eat processed meat (Luncheon-meat) samples were collected. Forty-four (12.6%) *K*. *pneumoniae* strains were isolated and bio-typed, where the majority were identified to belong to biotype B1. K1 and K2 serotypes were detected and strains were classified as hypermucoviscous *K*. *pneumoniae* (HVKP) and classic *K*. *pneumoniae* (CKP) (26 and 18 isolates, respectively). The isolates were resistant to several classes of β–lactam antibiotics, ceftazidim and cefotaxime (95.5%), cefoxitin (93.2%), ertapenem (90.9%) and amoxicillin-clavulanic acid (86.4%). They were classified as extended spectrum β–lactamases (ESBLs), AmpC or carbapenemase-producers phenotypically. Eighteen β-lactamase genes were investigated by PCR. The most prominent genes were *SHV* (63.6%), *TEM* (52.2%), *CTX-M15* (50%), *AMPC* (47.7%), *CIT-M* (45.5%) and *VIM* (43.2%). Co-detection of β–lactam resistance genes revealed 42 gene profiles. Twenty-four isolates had the complete efflux system (AcrAB-ToƖC). Besides, Integrons (I, II, III) were detected in 20 isolates. Molecular typing by ERIC-PCR showed high genetic diversity between isolates as 34 different patterns were identified. Overall, this study confirmed the hazards posed by the presence of multiple resistance genes in the same isolate and this should not be undervalued. Besides, the horizontal transfer of plasmid harboring resistance genes between isolates in food represents potential health risks for consumers in Egypt and so the control and inhibition plans are necessary.

## Introduction

*Klebsiella pneumoniae* is a facultative anaerobic *Enterobacteriaceae* that presents as normal microbiota of skin, mouth and intestine. However, it is also responsible for pneumonia, urinary, and lower biliary tract diseases [1], intra-abdominal, blood stream infections, meningitis, pyogenic liver abscess and prominent nosocomial infections. Although *K*. *pneumoniae* is not recognized as a food born pathogen; it can be isolated from raw vegetables, street foods [1], hospital waste water [2], fish and shrimps [3], farm raised chicken [4], and milk [5].

*K*. *pneumoniae* developed different resistance mechanisms to different classes of antimicrobial agents such as efflux pump, changing membrane permeability, production of inactivating enzymes, modification of target site, and acquisition of alternative metabolic pathway that is inhibited by antibiotics [6].

β–lactam is a large class of antibiotics that has different sub-classes including penicillins, cephalosporins, carbapenems, and monobactam. In Gram-negative (G-ve) bacteria, resistance to β–lactams is mediated by different strategies such as production of β–lactamases, efflux pumps, and alteration of penicillin binding proteins. The major mechanism involved in B lactam resistance is the production of β–lactamases [7]. Different β–lactamases have been discovered starting with broad spectrum β–lactamases now designated as extended spectrum β–lactamases (ESBLs), β–lactamases with reduced sensitivity to β–lactamase inhibitors, AmpC and β–lactamases that hydrolyze carbapenems. As β–lactamases are inducible enzymes, the presence of β–lactam antibiotics in food is considered a risk factor that generates the intrinsic resistance of the G-ve bacteria [7]. Consequently, the presence of antimicrobial resistant *K*. *pneumoniae* in food is a major health problem.

Bacterial efflux pump is an important system of resistance in G-ve bacteria. Antibiotics are ejected from the cell, thus decreasing antibiotic concentrations inside the cell. Efflux pumps are classified into five major families: the resistance-nodulation-division (RND) family, the major facilitator superfamily (MFS), the ATP (adenosine triphosphate)-binding cassette (ABC) superfamily, the small multidrug resistance (SMR) family, and the multidrug and toxic compound extrusion (MATE) family [8]. ERICs are repetitive intergenic sequences of the *Enterobacteriaceae* family comprising of 126 base pairs. ERIC-PCR fingerprinting methods are widely used for genetic typing of isolates as they produce fingerprints without the use of endonuclease enzyme activity. They can be used as genetic markers for genetic diversity among bacteria [9].

The present study aims to evaluate the frequency of *K*. *pneumoniae* in ready to eat processed meat, resistance to β–lactam antibiotics and to type the isolates serologically, and by molecular method (ERIC-PCR).

## Materials and methods

### Bacterial isolation and identification

Three hundred and fifty retail ready to eat bovine and/or chicken processed meat (Luncheon) were collected from different supermarkets in Mansoura city, Egypt as illustrated in (S1 Table). Each sample was collected in a sterile plastic bag stored in an icebox and processed within 4–6 hours as previously described [1].

These isolates were identified biochemically as Klebsiella species according to laboratory biochemical standards [10], where 44 *K*. *pneumoniae* and 3 *K*. *oxytoca* isolates were recovered. *K*. *pneumoniae* isolates were bio-typed according to the positive biochemical reactions according to (S2 Table) and used for further studies. The confirmed *K*. *pneumoniae* cultures were stored in -80°C for further studies.

The genomic DNA of all *K*. *pneumoniae* isolates was extracted as previously described [11]. The confirmation of isolates to be *K*. *pneumoniae* was done by PCR as illustrated by Osman *et al*., [12] using the primer sequence listed in Table 1. *K*. *pneumoniae* ATCC 5150 was used as a positive control.

**Table 1:** The sequence of primers used in the PCR amplification.

Target gene	Primer sequence 5՝-3՝	Annealing Temp (ᴼC)	Product size (bp)	Reference
*K*. *pneumoniae* 16S–23S ITSD	ATTTGAAGAGGTTGCAAACGAT TTCACTCTGAAGTTTTCTTGTGTTC	57	130	[12]
ESBL-genes				
TEM	GATCTCAACAGCGGTAAG CAGTGAGGCACCTATCTC	50	786	[11]
SHV	ACTATCGCCAGCAGGATC CAGTGAGGCACCTATCTC	53	356	[11]
CTX-M15	GTGATACCACTTCACCTC AGTAAGTGACCAGAATCAG	49	225	[11]
OXA-1-like	GGCACCAGATTCAACTTTCAAG GACCCCAAGTTTCCTGTAAGTG	60	564	[13]
GES	AGTCGGCTAGACCGGAAAG TTTGTCCGTGCTCAGGAT	60	399	[13]
PER	GCTCCGATAATGAAAGCGT TTCGGCTTGACTCGGCTGA	60	520	[13]
VEB	CATTTCCCGATGCAAAGCGT CGAAGTTTCTTTGGACTCTG	60	648	[13]
AmpC-genes				
AmpC	ACACGAGTTTGCATCGCCTG CTGAACTTACCGCTAAACAGTGGAAT	60	245	[14]
CIT-M	TGGCCAGAACTGACAGGCAAA TTCTCCTGAACGTGGCTGGC	60	462	[14]
FOX-1	GCAAACCAGCAATACCATCCA GCTCACCTTGTCATCCAGCTC	60	642	[14]
ACC-1	AGCTGTTATCCGTGATTACCTGTCT AGCGAACCCACTTCAAATAACG	60	248	[14]
ACT-1	CATGCTGGATCTGGCAACCT CTTCAGCGTCCAGCATTCCT	60	343	[14]
MOX	GCAACAACGACAATCCATCCT GGGATAGGCGTAACTCTCCCAA	60	895	[13]
Carbapenemase-genes				
OXA-48	GCTTGATCGCCCTCGATT GATTTGCTCCGTGGCCGAAA	57	281	[13]
NDM	GGTTTGGCGATCTGGTTTTC CGGAATGGCTCATCACGATC	52	621	[15]
IMP	TTGACACTCCATTTACDG GATYGAGAATTAAGCCACYCT	55	139	[13]
VIM	GATGGTGTTTGGTCGCATA CGAATGCGCAGCACCAG	55	390	[13]
KPC	CATTCAAGGGCTTTCTTGCTGC ACGACGGCATAGTCATTTGC	55	538	[13]
Efflux pump genes				
AcrA	GTGCCCAACAGTTTCTGATAACG GATGCTCTCAGGCAGCTTAGC	50	150	[16]
AcrB	TGAAAGATGCCATCAGCCGT ATTTTCACGAACGGCGTGGT	50	500	[16]
TOlC	AGAGTTTGATCMTGGCTCAG ACGAGCTGACGACARCCATG	48	700	[16]
Integrons				
Integron1	GGTCAAGGATCTGGATTTCG ACATGCGTGTAAATCATCGTC	60	436	[17]
Integron II	CACGGATATGCGACAAAAAGG TGTAGCAAACGAGTGACGAAATG	60	788	[17]
Integron III	AGTGGGTGGCGAATGAGTG TGTTCTTGTATCGGCAGGTG	60	600	**[17]**

### Serotyping

The tested isolates were serologically examined for the presence of capsular antigens K1 and K2 by Quelling test [18]. The antigen-antibody reactions were observed microscopically.

### String test

For the detection of mucoviscosity of the isolates, string test was performed as described previously [19]. The isolates were classified as hypermucoviscous *K*. *pneumoniae* (HVKP) isolates give positive results or classic *K*. *pneumoniae* (CKP) isolates give negative results.

### Determination of antibiogram

Antibiotic sensitivity testing against β-lactams antibiotics [Aztreonam (30), Ceftazidime (30), Cefotaxime (30), Cefoxitin (30), Ceftriaxone (30), Cefepime (30), Ertapenem (10), Imipenem(10), Meropenem (10) and amoxicillin-clavulanic acid (30)] was performed via the disc diffusion method as outlined by CLSI [20]. The MAR index (Multiple Antibiotic Resistance index) was calculated by dividing the number of antibiotics to which the specific isolate is drug-resistant by the total number of multiple antibiotics to which the specific isolate has been exposed [21].

### Phenotypic detection of β–lactamases enzymes

Phenotypic detection of ESBLs in *K*. *pneumoniae* isolates was performed by a double-disc synergy test (DDST) according to CLSI guidelines [20]. For AmpC β–lactamases activity, the test was conducted as reported by Barwa *et al*. [14]. The isolates showing distortion of the cefoxitin inhibition zone were considered as AmpC producers, while isolates with no distortion of the inhibition zone were identified as AmpC non-producers. Modified Hodges test (MHT) was used to test for carbapenemases enzymes according to CLSI [20].

### Molecular characterization of encoding genes

Eighteen uniplex PCR amplifications were done to detect the presence of different β–lactamases genes (*TEM*, *SHV*, *CTX-M15*, *OXA-1-like*, *OXA-48*, *GES*, *PER*, *VEB*, *AmpC*, *CIT-M*, *FOX-1*, *ACC-1*, *ACT-1*, *MOX* and *NDM*) [11, 13–15] beside efflux pump genes (*AcrA*, *AcrB and TOlC*) [16].

In addition, two multiplex PCR tests were performed. The first one was used to detect metallo-β–lactamases genes (*IMP*, *VIM*, and *KPC*) [13]. The second one detected the integron genes I, II, and III [17]. Table 1 showed the primer sequence, annealing temperature, and the product size of the used primers. The amplified PCR products were analyzed on 1.2% agarose gel stained with ethidium bromide, compared with 100 bp plus DNA ladder (Thermofisher Scientific) and scanned in gel documentation and analysis system (Model Gel Doc 1.4, 1189; AccuLab, USA).

### ERIC-PCR

Genomic DNA of each isolate was subjected to ERIC-PCR using the primers ERIC1 (5’-ATGTAAGCTCCTGGGGATTCAC-3’) and ERIC2 (5’-AAGTAAGTGACTGGGGTGAGCG-3’) and the PCR condition was followed as described elsewhere [1]. PCR products were analyzed by 2% agarose gel electrophoresis system. A similarity matrix was measured using Dice’s coefficient and the resultant dendrogram was made via the unweighted-pair group method with arithmetic averages (UPGMA).

### Statistical analysis

Graph-pad instate was used to analyze the results statistically. Fisher’s exact and Chi-square tests were used and P-value ≤ 0.05 was considered statistically significant.

## Results

### Biochemical and molecular identification of *K*. *pneumoniae*

In this study, 350 ready to eat processed meat specimens (luncheon meat) were collected, forty-four *K*. *pneumoniae* isolates were isolated, purified, and biochemically identified in addition to identification using PCR by amplification of *K*. *pneumoniae* 16S–23S ITSD with an incidence of 12.6%. Three different biotypes profiles were found among isolates using biochemical activities (S2 Table). The profile B1 was the predominant one, as it was found in 32 isolates (72.7%) with a P-value < 0.0001. The other profiles B3 and B4 were found in seven (15.9%) and five (11.3%) isolates, respectively. No isolates were classified as biotype B2 or B5.

### Phenotypic detection of capsular serotypes and hypermucoviscosity

*K*. *pneumoniae* serotypes was investigated using Quelling test. Two capsular types (K1 and K2) were identified among the tested isolates. Both serotypes account for 95.5% of isolates. 37 isolates (84%) were K1 with P-value < 0.0001 and only 5 isolates (11.4%) were K2. Besides that, two isolates (4.6%) were untypable (non K1/K2 serotype).

Hypermucoviscosity of isolates was measured using string test. It was found that 26 of the isolates were HVKP (P = 0.0109) while 18 isolates were CKP. The association of capsular serotypes and the hypermucoviscosity of isolates was investigated. It was found that K1 serotype was significantly associated with HVKP (P = 0.0364).

### Determination of antibiogram

The antimicrobial sensitivity test of *K*. *pneumoniae* isolates was measured using the disc diffusion method. The results showed that 42/44 (95.5%) isolates were resistant to cefoxitin and ceftazidim. In addition, 41(93.2%), 38(86.4%), and 40(90.9%) isolates were resistant to cefotaxime, amoxicillin-clavulanic acid, and ertapenem respectively. Moderate resistance level to aztreonam (63.6%), cefepime (43.2%), imipenem and meropenem (36.4%), and ceftriaxone (27.3%) was recorded. All isolates which showed resistance to imipenem and meropenem; were also resistant to ertapenem. Table 2 showed the antibiotic resistance pattern of the tested isolates. A5, A11, and A12 were the major antibiotypes being represented by 8, 6, and 7 isolates, respectively. Most of the isolates (93.2%) give MAR index of ≥ 0.5.

**Table 2:** Antibiotic resistance patterns of the tested *K*. *pneumoniae* isolates.

Antibiotype	No of antibiotics	MAR index	No. of isolates/pattern	Resistance pattern
A1	**10**	**1**	2	FOX, CAZ, CTX, CRO, FEP, AMC, ATM, ERT, IPM, MEM.
A2	**9**	**0.9**	1	FOX, CAZ, CTX, CRO, FEP, ATM, ERT, IPM, MEM.
A3			3	FOX, CAZ, CTX, FEP, AMC, ATM, ERT, IPM, MEM.
A4	**8**	**0.8**	3	FOX, CAZ, CTX, AMC, ATM, ERT, IPM, MEM.
A5			**8**	FOX, CAZ, CTX, CRO, FEP, AMC, ATM, ERT.
A6	**7**	**0.7**	1	FOX, CAZ, CTX, CRO, FEP, ATM, ERT.
A7			2	FOX, CAZ, CTX, FEP, AMC, ATM, ERT.
A8			4	FOX, CAZ, CTX, AMC, ERT, IPM, MEM.
A9	**6**	**0.6**	1	FOX, CAZ, CTX, FEP, ATM, MEM.
A10			3	FOX, CAZ, CTX, AMC, IPM, MEM.
A11			**6**	FOX, CAZ, CTX, AMC, ATM, ERT.
A12	**5**	**0.5**	**7**	FOX, CAZ, CTX, CRO, AMC, ERT.
A13	2	0.2	1	FOX, ERT.
A14	1	0.1	1	CAZ.
A15			1	ERT.

FOX: Cefoxitin; CAZ: ceftazidim; CTX: cefotaxime; CRO: ceftriaxone; FEP: cefepime; AMC: amoxicillin-clavulanic acid; ATM: aztreonam; ERT: ertapenem; IPM: imipenem; MEM: meropenem, MAR: multiple antibiotic resistance.

### Phenotypic detection of β-lactamases enzymes

In this study, the isolates were investigated for the production of ESBL, AmpC and carbapenemase enzymes phenotypically. The results revealed that 22, 21, and 16 isolates were ESBL-producers, AmpC-producers, and carbapenemase-producers, respectively. One isolate (4.8%) was classified as an ESBL and AmpC co-producer. Nine isolates were AmpC- and carbapenemase co-producers. Seven isolates were ESBL and carbapenemase co-producers. Two isolates were not classified as any of the aforementioned classes.

The correlation between the different β-lactamse classes and biotyping was studied. Biotype B1 was significantly present in ESBL-producers (P = 0.0455) and carbapenemase-producers (P<0.0001) while B2 was detected significantly in AmpC-producers (P = 0.0075). The serotype K1 was present in all classes, significantly (P<0.0001).

### Molecular characterization of resistance encoding genes and integrons

Examination of resistance encoding genes of the 44 isolates using PCR revealed that ESBLs genes, *SHV*, *TEM*, *CTX-M15*, *GES*, *VEB*, and *OXA-1-like* were harbored by 28, 23, 22, 17, eight, and three isolates, respectively. *PER* gene wasn’t harbored by any of the tested isolates. For AmpC genes, *AmpC*, *CIT-M*, *FOX-1*, and *ACT-1* were harbored by 21, 20, 11, and five isolates, respectively. *ACC-1* and *MOX* genes were not be detected in the tested isolates. Carbapenemases genes *VIM*, *NDM*, *OXA-48*, *IMP*, and *KPC* were detected in 18, 12, 10, 9, and five isolates, respectively. Therefore, 41, 35, and 31 isolates could be classified as ESBL, AmpC, and carbapenemase producers, respectively, as they harbored the genes specific for each class. For ESBL genes, the most predominant were *SHV*, *TEM*, and *CTX-M15* as they were harbored by 68.3%, 56.1%, and 53.7%, of isolates, respectively. For AmpC genes, *AmpC*, *CIT-M* were the most abundant genes being carried by 60% and 57.1%, respectively. Regarding Carbapenemases genes, *VIM* and *KPC* were the highest (58.1%) and the lowest (16.1%) genes detected, respectively. Among phenotypically classified ESBL-producers, detection of ESBL genes revealed that the most prominent genes were *SHV* (72.7%), *TEM* (59.1%), and *CTX-M15* (54.5%). In addition, they carried AmpC and carbapenemases genes (*AmpC*, *CIT-M*, *FOX-1*, *IMP*, *VIM*, *KPC*, *NDM*, and *OXA-48* in 8, 11, four, five, 11, four, five, and five isolates, respectively). Both *VEB* and *KPC* were significantly associated with ESBL-producers (P<0.0001 and P = 0.0143, respectively). Besides, it was found that *TEM* was co-detected significantly with *SHV* (P = 0.002).

Regarding phenotypically identified AmpC producers, AmpC genes (*AmpC*, *CIT-M*, *FOX-1*, and *ACT-1*) were detected in 61.9%, 47.6%, 38.1%, and 23.8%, respectively. The present study found that *ACT-1* and *FOX-1* genes were significantly presented in AmpC-producers (P<0.0016 and P = 0.0143, respectively). The other ESBL genes (*TEM*, *SHV*, *CTX-M15*, *OXA-1-Like*, and *GES*) were present in 11, 13, 10, one and 8 isolates, respectively, while carbapenemase genes (*IMP*, *VIM*, *KPC*, *NDM*, and *OXA-48*) were harbored by four, 8, one, 7, and five isolates, respectively. Moreover, AmpC gene was significantly associated with *CIT-M* (P = 0.039). Concerning the presence of carbapenemase genes among phenotypic carbapenemase-producers, *VIM* (62.5%), *NDM* (56.3%), and *IMP* (37.5%) were the most prevalent genes. ESBL genes (*TEM*, *SHV*, *CTX-M15 GES*, and *VEB*) were detected in 9, 10, 7, 6, and two of the tested isolates, respectively. On the other hand, AmpC genes (*AmpC*, *CIT-M*, *Fox-1*, and *ACT-1*) were found in 12, 8, three, and two isolates respectively. *IMP* gene was associated with *VIM* gene (P = 0.0324) and KPC was associated with *VIM* and *NDM* (P = 0.0455). In addition, *VIM* gene was correlated with HVKP (P = 0.0231) while *OXA-1-Like* and *ACT-1* genes were correlated with CKP isolates significantly.

The co-detection of β-lactam resistance genes in the tested isolates was shown in Table 3. For example, *TEM* was significantly associated with *GES*, *AmpC*, *FOX-1*, *ACT-1*, and *IMP*. *CIT-M* was co-detected significantly with *FOX-1* and *VIM*. Besides, *IMP* was significantly associated with *VIM* and *CTX-M15*.

**Table 3:** Co-detection rates of β-lactam resistance encoding genes amongst 44 *K*. *pneumoniae* isolates.

	Observed frequency (%)													
Resistance encoding genes														
	*TEM*	*SHV*	*CTX-M15*	*OXA-1-like*	*GES*	*VEB*	*AmpC*	*CIT-M*	*FOX-1*	*ACT-1*	*OXA-48*	*NDM*	*IMP*	*VIM*
*TEM*														
*SHV*	61													
*CTX-M15*	43	46												
*OXA-1-like*	9	7	5											
*GES*	39***	39	50**	33										
*VEB*	17	18	22	33*	12									
*AmpC*	39**	32***	55	67*	47	50***								
*CIT-M*	52	50	55***	67**	53	50	52							
*FOX-1*	35*	27	32	33	29	38	29	40***						
*ACT-1*	17*	17*	9	33***	6	0***	14	10	0***					
*OXA-48*	90	18*	22	33	30	**20**	50	60*	40*	10				
*NDM*	22	29	23	0***	41**	0***	33	25	25	20	30			
*IMP*	35***	27	23	33	12**	13*	29	30	25	0***	40***	25		
*VIM*	43	46	22***	33	47	38	48	60***	67***	20***	50	50	70***	
*KPC*	9	7	14*	0**	24***	13	14*	10	8	0**	10	17*	10	75***

***P, 0.0001

**P, 0.001

*P, 0.05, Numbers in Bold are statistically significant associations.

Investigation of the gene profile of the tested isolates revealed that there were 42 different gene profiles among the 44 isolates as illustrated in Table 4. There was only one profile (Pr1) carried by two isolates while each of the other profiles was represented by one isolate. Two isolates harbored nine genes, 10 isolates carried four genes and five and six genes were carried by eight and six isolates, respectively. Resistance genes profiles were unique as each profile was represented by only one isolate.

**Table 4:** Relationship between ERIC typing and β-lactam resistance encoding genes profiles.

ERIC pattern	No. of genes	β-lactam resistance encoding genes profile	β-lactam resistance encoding genes															No. of total isolates
			*TEM*	*SHV*	*CTX-M15*	*OXA-1-like*	*GES*	*VEB*	*AmpC*	*CIT-M*	*FOX-1*	*ACT-1*	*OXA-48*	*NDM*	*IMP*	*VIM*	*KPC*	
1	1	Pr1	**-**	**-**	**-**	**-**	**-**	**-**	**+**	**-**	**-**	**-**	**-**	**-**	**-**	**-**	**-**	**2**
2																		
6	2	Pr2	**-**	**+**	**-**	**-**	**-**	**-**	**-**	**-**	**+**	**-**	**-**	**-**	**-**	**-**	**-**	**1**
13		Pr3	**-**	**-**	**+**	**-**	**-**	**-**	**+**	**-**	**-**	**-**	**-**	**-**	**-**	**-**	**-**	**1**
5	3	Pr4	**-**	**+**	**+**	**-**	**-**	**-**	**-**	**-**	**-**	**+**	**-**	**-**	**-**	**-**	**-**	**1**
14		Pr5	**-**	**-**	**-**	**-**	**-**	**+**	**+**	**-**	**-**	**-**	**-**	**-**	**-**	**+**	**-**	**1**
28		Pr6	**+**	**-**	**-**	**-**	**-**	**+**	**-**	**-**	**-**	**-**	**-**	**-**	**-**	**+**	**-**	**1**
24		Pr7	**-**	**+**	**-**	**-**	**-**	**-**	**+**	**-**	**-**	**-**	**-**	**+**	**-**	**-**	**-**	**1**
34		Pr8	**+**	**-**	**-**	**-**	**-**	**-**	**-**	**-**	**-**	**-**	**+**	**+**	**-**	**-**	**-**	**1**
1	4	Pr9	**+**	**-**	**-**	**-**	**+**	**-**	**-**	**-**	**+**	**-**	**-**	**-**	**-**	**+**	**-**	**1**
7		Pr10	**-**	**+**	**+**	**-**	**+**	**-**	**+**	**-**	**-**	**-**	**-**	**-**	**-**	**-**	**-**	**1**
9		Pr11	**+**	**-**	**+**	**-**	**-**	**-**	**+**	**-**	**-**	**-**	**-**	**-**	**+**	**-**	**-**	**1**
11		Pr12	**-**	**+**	**+**	**-**	**+**	**-**		**+**	**-**	**-**	**-**	**-**	**-**	**-**	**-**	**1**
16		Pr13	**-**	**+**	**+**	**-**	**+**	**-**	**-**	**-**	**-**	**-**	**-**	**-**	**-**	**+**	**-**	**1**
18		Pr14	**+**	**+**	**+**	**-**	**-**	**-**	**-**	**+**	**-**	**-**	**-**	**-**	**-**	**-**	**-**	**1**
19		Pr15	**-**	**+**	**+**	**-**	**-**	**+**	**-**	**+**	**-**	**-**	**-**	**-**	**-**	**-**	**-**	**1**
27		Pr16	**+**	**+**	**-**	**-**	**-**	**+**	**-**	**+**	**-**	**-**	**-**	**-**	**-**	**-**	**-**	**1**
33		Pr17	**+**	**+**	**+**	**-**	**-**	**-**	**-**	**-**	**-**	**+**	**-**	**-**	**-**	**-**	**-**	**1**
untypable		Pr18	**+**	**-**	**+**	**-**	**-**	**-**	**-**	**-**	**+**	**-**	**+**	**-**	**-**	**-**	**-**	**1**
3	5	Pr19	**+**	**+**	**+**	**-**	**-**	**-**	**-**	**+**	**-**	**-**	**-**	**-**	**+**	**-**	**-**	**1**
13		Pr20	**-**	**-**	**+**	**-**	**+**	**-**	**+**	**+**	**-**	**-**	**-**	**+**	**-**	**-**	**-**	**1**
14		Pr21	**+**	**+**	**-**	**-**	**+**	**+**	**-**	**-**	**-**	**-**	**+**	**-**	**-**	**-**	**-**	**1**
15		Pr22	**+**	**+**	**-**	**+**	**-**	**-**	**-**	**-**	**-**	**-**	**-**	**-**	**+**	**+**	**-**	**1**
21		Pr23	**+**	**+**	**-**	**-**	**-**	**-**	**+**	**-**	**-**	**+**	**-**	**+**	**-**	**-**	**-**	**1**
26		Pr24	**+**	**+**	**+**	**-**	**+**	**-**	**-**	**-**	**-**	**-**	**-**	**+**	**-**	**-**	**-**	**1**
29		Pr25	**-**	**+**	**-**	**-**	**+**	**-**	**-**	**-**	**-**	**-**	**-**	**+**	**-**	**+**	**+**	**1**
31		Pr26	**-**	**+**	**-**	**-**	**-**	**-**	**+**	**-**	**-**	**-**	**-**	**+**	**+**	**+**	**-**	**1**
1	6	Pr27	**+**	**+**	**-**	**-**	**+**	**-**	**-**	**+**	**+**	**-**	**-**	**-**	**-**	**+**	**-**	**1**
4		Pr28	**+**	**+**	**-**	**-**	**-**	**-**	**-**	**+**	**-**	**-**	**+**	**-**	**+**	**+**	**-**	**1**
8		Pr29	**-**	**+**	**+**	**-**	**+**	**-**	**+**	**+**	**-**	**-**	**-**	**-**	**-**	**+**	**-**	**1**
13		Pr30	**+**	**+**	**-**	**-**	**+**	**-**	**-**	**-**	**-**	**-**	**+**	**+**	**+**	**-**	**-**	**1**
25		Pr31	**+**	**+**	**-**	**-**	**-**	**-**	**+**	**+**	**-**	**+**	**-**	**-**	**-**	**+**	**-**	**1**
22		Pr32	**+**	**+**	**-**	**-**	**-**	**-**	**-**	**+**	**+**	**-**	**-**	**-**	**+**	**+**	**-**	**1**
1	7	Pr33	**-**	**+**	**+**	**+**	**-**	**+**	**+**	**+**	**+**	**-**	**-**	**-**	**-**	**-**	**-**	**1**
2		Pr34	**+**	**-**	**+**	**-**	**+**	**+**	**+**	**-**	**+**	**-**	**-**	**-**	**-**	**-**	**+**	**1**
17		Pr35	**-**	**+**	**+**	**-**	**+**	**-**	**-**	**+**	**+**	**-**	**-**	**+**	**-**	**+**	**-**	**1**
30		Pr36	**+**	**-**	**-**	**+**	**+**	**-**	**+**	**+**	**-**	**+**	**+**	**-**	**-**	**-**	**-**	**1**
32		Pr37	**-**	**+**	**-**	**-**	**-**	**-**	**+**	**+**	**+**	**-**	**+**	**+**	**-**	**+**	**-**	**1**
10	8	Pr38	**+**	**-**	**+**	**-**	**-**	**-**	**+**	**+**	**+**	**-**	**+**	**-**	**+**	**+**	**-**	**1**
12		Pr39	**+**	**-**	**+**	**-**	**+**	**-**	**+**	**+**	**+**	**-**	**-**	**+**	**-**	**+**	**-**	**1**
20		Pr40	**-**	**-**	**+**	**-**	**+**	**-**	**+**	**+**	**-**	**-**	**-**	**+**	**+**	**+**	**+**	**1**
3	9	Pr41	**+**	**+**	**+**	**-**	**+**	**-**	**+**	**+**	**-**	**-**	**+**	**-**	**-**	**+**	**+**	**1**
23		Pr42	**+**	**-**	**+**	**-**	**-**	**+**	**+**	**+**	**+**	**-**	**+**	**-**	**+**	**+**	**-**	**1**

Pr: profile.

For efflux pump genes, *AcrA*, *AcrB* and *TOlC* were amplified in 42 (95.5%), 28(63.6%) and 37(84.1%) isolates, respectively. Twenty-four isolates harbored the three genes. Six of them were ESBL-producers, four isolates were ESBL and carbapenemase-coproducers, five were AmpC-producers and eight were AmpC and carbapenemase-coproducers. Isolate No. 39 (ESBL and AmpC-coproducer) harbored the three efflux genes.

Investigation of the distribution of integrons between isolates revealed that integrons (I, II, III) were detected in 20/44 isolates. Eleven, two, and four isolates were found to carry Int I, Int II, and Int III, respectively. Two isolates harbored both Int I and Int III and one isolate carried Int II and Int III. ESBL-producers were shown to be significantly associated with Int I (P = 0.033). In AmpC-producers, only 9/21 of isolates harbored Int I (three isolates), Int III (three isolates), each of Int II, Int I+Int III, and Int II+Int III was carried by one isolate. Six isolates in carbapenemase-producers harbored Int I (two isolates), Int III (three isolates), and Int II+ Int III (one isolate). Int I and Int III were present significantly in K1 serotyped isolates (P = 0.02 and 0.07, respectively). It was found that isolates carrying integrons were resistant to 5 or more antimicrobials (P< 0.0001), harbored 5 or more of resistance genes (P = 0.0256), and carried the complete efflux system (P = 0.0038).

### ERIC

**Fig 1:**
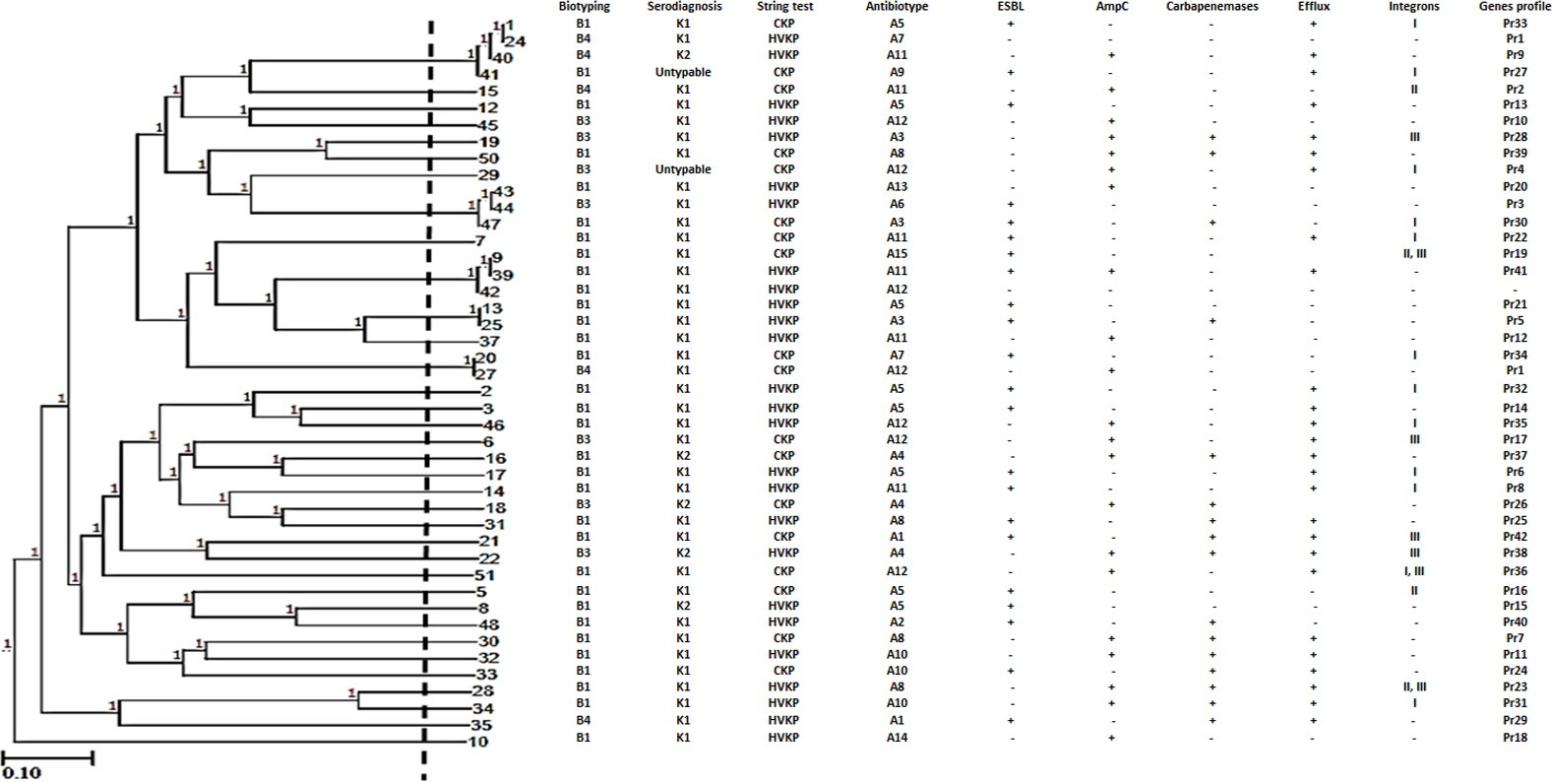
Genotyping of *K*. *pneumoniae* isolates using ERIC-PCR method. **The dendrogram was constructed using ERIC-PCR patterns of *K*. *pneumoniae* isolates.** Similarity clustering analysis was performed using the UPGMA and Dice coefficient. The dashed line is a hypothetical line showing 85% similarity. Biotyping, serodiagnosis, hypermucoviscosity, ESBL-producers, AmpC-producers, Carbapenemase-producers, efflux system, integrons, and genes profile among *K*. *pneumoniae* isolates were reported. B: biotype, CKP: classical *K*. *pneumoniae*, HVKP: hypermucoviscous *K*. *pneumoniae*, U: untypable, Pr: profile, ERIC-PCR, enterobacterial repetitive intergenic consensus-PCR. UPGMA, unweighted-pair group method with arithmetic averages.

ERIC molecular typing gave high diversity among the tested isolates. As seen in Fig 1, ERIC-PCR classified the isolates into 34 different patterns. Pattern 1 included four isolates, patterns 3 and 13 each enclosed three isolates, and patterns 2 and 14 each contained two isolates while the other patterns, each one represented by one isolate. The number of bands varied between one and 12 bands, which ranged between 100 and >1500 bp. Distribution of ERIC patterns among isolates harboring β-lactam resistance encoding genes was illustrated in Table 3 where 42 different resistance gene profiles were distributed among 34 ERIC patterns that represent all tested *K*. *pneumoniae* isolates, where ERIC pattern 1 and 2 have the same gene profile (Pr1).

## Discussion

*K*. *pneumoniae* is an opportunistic pathogen that is found not only in clinical specimens but also in food. Foodborne pathogens are widely distributed but research about *K*. *pneumoniae* as a food pathogen is infrequent. Recently, *K*. *pneumoniae* is the main cause of foodborne outbreaks in different countries [1]. In this study, *K*. *pneumoniae* isolates were isolated from ready to eat processed meat (Luncheon) with an incidence of 12.6% which indicates that contaminated food with *K*. *pneumoniae* is common in Egypt. Similar results were reported in china where the incidence of *K*. *pneumoniae* in 998 food samples was 9.9% [4]. Another study conducted in Spain isolated only 9 strains (5.6%) of *K*. *pneumoniae* from 160 vegetables [22].

One of the important virulence factors of *K*. *pneumoniae* is the production of a capsule that gives it a mucoid appearance on an agar plate beside that it protects it against phagocytosis and serum bactericidal effect [1]. In addition, classifying *K*. *pneumoniae* isolates as virulent ones are associated with serotypes K1 and K2 [23, 24]. Two capsular types (K1 and K2) were identified by Quelling test. Two isolates (4.6%) were untypable (non K1/K2 serotype). Yu *et al*. had described that among 50 *K*. *pneumoniae* isolates, 26 (52%) were K1, 14 (28%) were K2 and 10 (20%) isolates were non K1/K2 serotype [20]. Other studies reported different prevalence rates for K1 and K2, where four capsular types (K1, K2, K20, and K54) were identified in 8 isolates only and the rest of the isolates were non K1/K2 serotypes [1]. Besides that, another study illustrated that K1 and K2 serotypes were found in 28.5% and 7.14% of the tested *K*. *pneumoniae* isolates, respectively [25].

Another virulence factor of *K*. *pneumoniae*, hypermucoviscosity, was measured using the string test. The results showed that 59% of isolates were HVKP and 41% of isolates were CKP. In contrast, Gharrah *et al*. [11] found that 33% of isolates were HVKP. Diverse capsular ingredients and an increased amount of capsular material have been described in hypervirulent *K*. *pneumoniae* isolates [26]. Similar to other studies that reported that HVKP isolates are associated with K1 or K2 serotype [23], the current results illustrated that K1 and K2 serotypes were found as both HVKP (59.1%; 23 and 3 isolates respectively) and CKP (36.4%; 14 and 2 isolates respectively) but significantly associated with HVKP (P = 0.0018). However, little work elucidating the role of the hypermucoviscous (HMV) phenotype in the pathogenicity of *K*. *pneumoniae* exists [11].

The extensive use of antimicrobials in the ecosystems resulted in the emergence of antimicrobial-resistant *K*. *pneumoniae*. In the current study, most of the isolates (93.2%) were resistant to cefotaxime. These isolates usually produce ESBL and show resistance to other β-lactam antibiotics [27, 28]. In addition, the studied isolates show high resistance to ceftazidim, cefoxitin amoxicillin-clavulanic acid, and ertapenem, and moderate resistance level to aztreonam, cefepime, imipenem, meropenem, and ceftriaxone. There was a wide assortment of resistance patterns among isolates, where 15 antibiotypes were found. The predominant antibiotype was A5 which includes isolates resistant to 8 antimicrobials. 93.2% of isolates were resistant to five or more antimicrobials with MAR index ≥0.5. The presence of such resistant isolates in food represents a risk factor to the food consumers as they are considered as high-risk sources of antimicrobial contamination, also, they cause infections that can’t be treated by these agents and result in an increase in the morbidity and mortality rates. The isolates that show resistance to ertapenem, meropenem, and imipenem (36.4%) are more likely to produce carbapenemase enzymes. These findings are contrary to what was reported by another study which found that 3.2% of isolates were resistant to amoxicillin-clavulanic acid, cefotaxime, and cefoxitin. Besides that, resistance to ceftriaxone was only 1.6% [1]. The high rates of antimicrobial resistance detected in this study can be attributed to the lack of strict policies that govern the use of antibiotics in Egypt.

In this study 22 isolates were ESBL-producers, 21 isolates were AmpC-producers and 16 isolates were carbapenemase-producers. Two isolates were not categorized as any of the previous classes. The percentage of *K*. *pneumoniae* isolates that produce ESBLs differs between countries. Their percent in Arabian countries is high (62.5% and 50% of isolates) as reported by Aljanaby and Alhasani [29] and Gharrah *et al*. [11], respectively. This is similar to the present results as 50% of isolates were categorized as ESBL-producers. This may have a significant effect on the treatment strategies using β-lactam antibiotics which increase the morbidity and mortality among food consumers. AmpC enzymes are β-lactamases that hydrolyze penicillins, cephalosporins, and cephamycins with low hydrolysis rates for cefepime, cefpirome, and carbapenems [30]. They aren’t inactivated by available β-lactamases inhibitors in contrast to ESBLs [31]. The present results showed that 47.7% of isolates were AmpC-producers with 4.8% classified as ESBL and AmpC co-producer. Barwa *et al*. showed that 31.6% of isolates were AmpC-producers [14], moreover, 65 *K*. *pneumoniae* isolates were ESBL-producers and 7.7% of them were AmpC-producers as reported by Zorgani *et al*. [32].

Among used antibiotics, carbapenem is considered the choice for the treatment of critical infections due to multidrug-resistant *K*. *pneumoniae*. However, there is an increasing incidence of carbapenem resistance through *K*. *pneumoniae* isolates in many countries due to the extensive use of the carbapenems [33]. In the present work, 38.6% of isolates were found to be carbapenemase-producers, 56.3% of them were AmpC-coproducer while the rest were ESBL-producers too. Japoni-Nejad *et al*. showed that 12 isolates were carbapenemases-producers and 8 of them were AmpC-producers [34]. Besides, Yazgan *et al*. showed that 51% of collected *K*. *pneumoniae* were ESBL-producers, 49% of them were carbapenemase-producers [8]. These results may be attributed to the excessive use of these antibiotics. There is a significant increase in carbapenem resistance *K*. *pneumoniae* isolates in Egypt as it increased from 13.9% to 44.4% [25, 35]. Several mechanisms are responsible for this type of resistance including AmpC or ESBL production with porin loss, carbapenemase production, or metallo-β-lactamase production [36].

All phenotypic methods used in this study detects isolates as positive ESBL, AmpC, or carbapenemase producer but they were unable to differentiate the different types or families of each class. Therefore, it is necessary to use other techniques such as PCR for confirmation of phenotypic results and discrimination of different genes. Examination using PCR classified 41, 35, and 31 isolates as ESBL, AmpC, and carbapenemase producers, respectively. Sutandhio *et al*. showed that 12 isolates harbored carbapenemase genes but only 6 isolates of them were Modified Hodges test positive [37]. Another study showed that 37.5% of *K*. *pneumoniae* isolates were ESBL or AmpC-producers phenotypically while 43% of them were ESBL only, 11% were AmpC and 36% were ESBL and AmpC genotypically [38]. Similar results were observed in other studies [14, 39]. This variation between phenotypic and PCR results may be because these genes are present but are not usually expressed [14]. Therefore, not all isolates detected as ESBL, AmpC, and carbapenemases producers by PCR can be detected by phenotypic methods.

PCR is considered the gold standard method for the detection of different classes of β-lactamase enzymes [40]. The PCR amplification of β-lactam resistance genes was performed on all isolates. For ESBL genes, the most predominant were *SHV*, *TEM*, and *CTX-M15* as harbored by 63.6%, 52.3%, and 50%, of isolates, respectively. Similarly, Hasani *et al*. illustrated that SHV gene was the predominant gene (80.9%) in the isolates followed by CTX-M and TEM (73% and 58%, respectively) [41]. For AmpC genes, *AmpC*, *CIT-M* were the most abundant genes as were carried by 47.7% and 45.5%, respectively. In contrast, Ghonaim and Moaety showed that *CIT* and *MOX* genes were present in 18.9% and 6.1%, respectively, and *ACC*, *FOX*, and *ACT* genes were not detected [42]. In Carbapenemases genes, *VIM* and *KPC* were the highest (43.2%) and the lowest (9.1%) genes detected respectively. In contrary to other studies that reported that *OXA-48*, *NDM*, and *VIM* were the predominant genes in this order [26]. ESBL and AmpC-coproducer (isolate No. 39) harbors 9 genes (Pr41). For efflux pump genes, *AcrA*, *AcrB* and *TOlC* were amplified in 95.5%, 63.6% and 84.1% of isolates, respectively. The coexistence of several genes of ESBL, AmpC, and carbapenemases in the same isolate revealed serious epidemiological, clinical and public health threats.

The association between β-lactam resistance encoding genes was investigated amongst the 44 *K*. *pneumoniae* isolates as illustrated in Table 3 to determine if there was a non-random association between genes that may be due to genes co-location. Some combinations of β-lactam resistance encoding genes were detected significantly. In contrast to our results, Lalzampuia *et al*., [43] found that for eight isolates, 7 harbored *CTX-M-1* gene and/or *TEM* gene. The *CTX-M* and *TEM* were the most common gene combinations (33.33%) in *E*. *coli* isolates from broiler farms in the Philippines [44]. Benmahmod *et al*. reported that one isolate had both *IMP* and *NDM*, and one isolate co-carried *IMP* and *VIM* [45].

The investigation of the β-lactam resistance genes profile revealed that 42 different profiles were distributed among 44 *K*. *pneumoniae* isolates. The gene profile of isolates showed that there is a great genetic diversity between isolates and that the plasmids encoding for β-lactamases can be easily spread by horizontal transfer among *Enterobacteriaceae* including *K*. *pneumoniae* [33].

Efflux pump systems in *K*. *pneumoniae* include AcrAB and mdtK systems, that belong to (RND) and (MATE) family efflux pumps, respectively. The AcrAB-TolC pump is composed of an outer-membrane channel (TolC), a secondary transporter located in the inner membrane (AcrB), and a periplasmic component (AcrA) [25, 46]. In this study, the Efflux pump system consisting of *AcrAB-ToƖC* was determined by PCR. Twenty-four isolates (54.5%) harbored the three genes. On the other hand, 20 isolates were missing either the AcrA or AcrB efflux pump or the ToƖC outer membrane protein. Other studies showed that 82.14% of isolates harbored *AcrAB* while only 5 isolates showed an incomplete AcrAB efflux system [25]. In addition, another study showed that 100% of isolates carried *AcrAB* while 96% carried *ToƖC* genes [23]. Multidrug efflux system (AcrAB- ToƖC) is responsible for the resistance of *K*. *pneumoniae* to β-lactam, tetracyclines, and quinolones [23].

Investigation of integrons distribution among isolates revealed that Integrons (I, II, III) were detected in 45.5% of isolates, of which 55%, 10%, and 20% of isolates carried Int I, Int II, and Int III, respectively. Both Int I and Int III were co-founded in 20% of isolates and 10% of isolate co-carried Int II and Int III. A study conducted by Sedighi *et al*. showed that Int I was present in 8% of isolates while Int II and Int III were absent from all isolates [47]. Rezai *et al*. reported that Int I was detected in 79.3% while Int II was harbored by 10.3% of isolates[48]. ESBL-producers were significantly associated with Int I (P = 0.033), similar to what reported by Elsherif *et al*., as Int 1 was associated with CTX-M gene (P = 0.039) [49]. In contrary to what reported about the association of Int III with *GES* gene [49], current results showed that they coexist only in one isolate out of seven Int III positive isolates. Additionally, *IMP* gene coexisted with Int III in 42.9% of isolates which was unlike the study conducted by Elsherif *et al*. who reported that Int III is of low occurrence with IMP gene [49].

In this study, the characterization of *NDM*-encoding isolates was examined where NDM can break down all the β-lactam antibiotics except aztreonam. This gene is located on a transmissible plasmid and is associated with other resistant genes. This may result in a broad drug resistance, which makes the treatment options limited [33]. Herein, these isolates were significantly classified as K1 (P = 0.0011) and B1 (P<0.0001). Half of them were classified as HVKP. Only four isolates harbored integrons, three of them carry Int I and one isolate carry IntII+IntIII. Nine out of the 12 isolates were confirmed as carbapenemase-producers by phenotypic and PCR methods, while three isolates were identified only by PCR. This may be attributed to one of the Modified Hodges test drawbacks in that it gives false-negative results for NDM producers [50]. *NDM* gene was present as sole carbapenemase gene in four (33%) isolates, significantly associated with *VIM* gene in six isolates (P<0.0001), with *IMP* gene in three isolates (25%), with *OXA-48* in four isolates (33%) and with *KPC* in two isolates (16.7%)(P = 0.0249), in addition, it was significantly associated with ESBL genes (*GES* and *OXA-1-like*) with P = 0.0011 and <0.0001, respectively. Khalil *et al*. also reported the association between NDM and other carbapenemase genes as *KPC*, *IMP*, *VIM*, and *OXA-48* where they were detected in NDM-1 producing *K*. *pneumoniae* with the frequency of 80%, 30%, 50%, and 30% respectively [33]. Eight isolates harbored the three efflux genes (66.7%) with P = 0.0214. Although *NDM* is a broad spectrum carbapenemase that can inactivate β-lactam except for aztreonam [25], four isolates in this study showed resistance to aztreonam and harbored *NDM* indicating that a new antimicrobial resistance pattern in Egyptian isolates may exist.

Recently, ERIC-PCR is one of the most potent tools used for the analysis of genetic relatedness in *K*. *pneumoniae* [33]. ERIC molecular typing results gave high diversity among the tested isolates as it classified the isolates into 34 different patterns. The association between phenotypic and molecular typing gave no clear relation between isolates except in the case of isolates No. 47 and 41among ESBL-producers and isolates No. 22 and 19 among carbapenemase-producers. The relation between ERIC patterns and β-lactam resistance encoding genes profiles was studied where 42 different resistance gene profiles were distributed among 34 ERIC patterns that represent all tested *K*. *pneumoniae* isolates. Similar results were reported by Kazemian *et al*. [51] Our results indicated the significance and convenience of ERIC-PCR and β-lactam resistance encoding genes profiles in differentiating isolates based on genetic relatedness. The diversity in our isolates represented a problem in the treatment of *K*. *pneumoniae* infections. Similar genetic diversity represented by ERIC was described in previous studies [9, 33].

In conclusion, the present study indicated that food (ready to eat processed meat; Luncheon) is a good reservoir of resistant *K*. *pneumoniae* isolates. This represents a public health problem and good control of the emergence and the transmission of these isolates is needed. This can be done by developing more prevention strategies on making and selling this food in supermarkets.

## Supporting information

S1 Table The complete list of the exact sources of all samples used in the study. (DOCX)

S2 Table Classification of *K*. *pneumoniae* according to biochemical reactions. (DOCX)
