# Supplementary material for: Characterization of β-lactam resistance in K. pneumoniae associated with ready-to-eat processed meat in Egypt
Source: PLoS One. 2020 Sep 3;15(9):e0238747. doi: 10.1371/journal.pone.0238747 (PMC7470258; doi:10.1371/journal.pone.0238747)
Supplement: S1 Table — (DOCX) [file pone.0238747.s001.docx]

**S1 Table: The complete list of the exact sources of all samples used in the study.**

| Sample No. | Source | | |
| --- | --- | --- | --- |
| 1 | Beef luncheon meat | Supplier 1 | Supermarket 1 |
| 2 | Chicken luncheon meat | Supplier 1 | Supermarket 1 |
| 3 | Turkey-Chicken Luncheon Meat | Supplier 1 | Supermarket 1 |
| 4 | Beef luncheon meat | Supplier 2 | Supermarket 1 |
| 5 | Chicken luncheon meat | Supplier 2 | Supermarket 1 |
| 6 | Turkey-Chicken Luncheon Meat | Supplier 2 | Supermarket 1 |
| 7 | Beef luncheon meat | Supplier 3 | Supermarket 1 |
| 8 | Chicken luncheon meat | Supplier 3 | Supermarket 1 |
| 9 | Turkey-Chicken Luncheon Meat | Supplier 3 | Supermarket 1 |
| 10 | Beef luncheon meat | Supplier 4 | Supermarket 1 |
| 11 | Chicken luncheon meat | Supplier 4 | Supermarket 1 |
| 12 | Beef luncheon meat | Supplier 5 | Supermarket 1 |
| 13 | Chicken luncheon meat | Supplier 5 | Supermarket 2 |
| 14 | Beef luncheon meat | Supplier 6 | Supermarket 1 |
| 15 | Chicken luncheon meat | Supplier 6 | Supermarket 1 |
| 16 | Beef luncheon meat | Supplier 7 | Supermarket 1 |
| 17 | Chicken luncheon meat | Supplier 7 | Supermarket 1 |
| 18 | Beef luncheon meat | Supplier 8 | Supermarket 1 |
| 19 | Chicken luncheon meat | Supplier 8 | Supermarket 1 |
| 20 | Beef luncheon meat | Supplier 2 | Supermarket 2 |
| 21 | Chicken luncheon meat | Supplier 2 | Supermarket 2 |
| 22 | Beef luncheon meat | Supplier 2 | Supermarket 3 |
| 23 | Chicken luncheon meat | Supplier 2 | Supermarket 3 |
| 24 | Beef luncheon meat | Supplier 3 | Supermarket 4 |
| 25 | Chicken luncheon meat | Supplier 3 | Supermarket 4 |
| 26 | Beef luncheon meat | Supplier 4 | Supermarket 4 |
| 27 | Chicken luncheon meat | Supplier 4 | Supermarket 4 |
| 28 | Beef luncheon meat | Supplier 5 | Supermarket 4 |
| 29 | Chicken luncheon meat | Supplier 5 | Supermarket 4 |
| 30 | Beef luncheon meat | Supplier 1 | Supermarket 5 |
| 31 | Chicken luncheon meat | Supplier 1 | Supermarket 5 |
| 32 | Turkey-Chicken Luncheon Meat | Supplier 1 | Supermarket 5 |
| 33 | Beef luncheon meat | Supplier 2 | Supermarket 5 |
| 34 | Chicken luncheon meat | Supplier 2 | Supermarket 5 |
| 35 | Turkey-Chicken Luncheon Meat | Supplier 2 | Supermarket 5 |
| 36 | Beef luncheon meat | Supplier 3 | Supermarket 5 |
| 37 | Chicken luncheon meat | Supplier 3 | Supermarket 5 |
| 38 | Turkey-Chicken Luncheon Meat | Supplier 3 | Supermarket 5 |
| 39 | Beef luncheon meat | Supplier 7 | Supermarket 6 |
| 40 | Beef luncheon meat | Supplier 8 | Supermarket 7 |
| 41 | Beef luncheon meat | Supplier 6 | Supermarket 8 |
| 42 | Beef luncheon meat | Supplier 3 | Supermarket 9 |
| 43 | Beef luncheon meat | Supplier 5 | Supermarket 10 |
| 44 | Beef luncheon meat | Supplier 4 | Supermarket 11 |
| 45 | Chicken luncheon meat | Supplier 4 | Supermarket 11 |
| 46 | Beef luncheon meat | Supplier 1 | Supermarket 12 |
| 47 | Chicken luncheon meat | Supplier 1 | Supermarket 12 |
| 48 | Turkey-Chicken Luncheon Meat | Supplier 1 | Supermarket 12 |
| 49 | Beef luncheon meat | Supplier 2 | Supermarket 12 |
| 50 | Chicken luncheon meat | Supplier 2 | Supermarket 12 |
| 51 | Turkey-Chicken Luncheon Meat | Supplier 2 | Supermarket 12 |
| 52 | Beef luncheon meat | Supplier 3 | Supermarket 12 |
| 53 | Chicken luncheon meat | Supplier 3 | Supermarket 12 |
| 54 | Turkey-Chicken Luncheon Meat | Supplier 3 | Supermarket 12 |
| 55 | Beef luncheon meat | Supplier 4 | Supermarket 12 |
| 56 | Chicken luncheon meat | Supplier 4 | Supermarket 12 |
| 57 | Beef luncheon meat | Supplier 6 | Supermarket 12 |
| 58 | Chicken luncheon meat | Supplier 6 | Supermarket 12 |
| 59 | Beef luncheon meat | Supplier 8 | Supermarket 12 |
| 60 | Chicken luncheon meat | Supplier 8 | Supermarket 12 |
| 61 | Beef luncheon meat | Supplier 1 | Supermarket 13 |
| 62 | Beef luncheon meat | Supplier 6 | Supermarket 14 |
| 63 | Beef luncheon meat | Supplier 7 | Supermarket 15 |
| 64 | Beef luncheon meat | Supplier 1 | Supermarket 16 |
| 65 | Beef luncheon meat | Supplier 1 | Supermarket 17 |
| 66 | Beef luncheon meat | Supplier 3 | Supermarket 18 |
| 67 | Beef luncheon meat | Supplier 2 | Supermarket 19 |
| 68 | Beef luncheon meat | Supplier 5 | Supermarket 20 |
| 69 | Beef luncheon meat | Supplier 8 | Supermarket 21 |
| 70 | Beef luncheon meat | Supplier 1 | Supermarket 22 |
| 71 | Chicken luncheon meat | Supplier 1 | Supermarket 22 |
| 72 | Turkey-Chicken Luncheon Meat | Supplier 1 | Supermarket 22 |
| 73 | Beef luncheon meat | Supplier 2 | Supermarket 22 |
| 74 | Chicken luncheon meat | Supplier 2 | Supermarket 22 |
| 75 | Turkey-Chicken Luncheon Meat | Supplier 2 | Supermarket 22 |
| 76 | Beef luncheon meat | Supplier 3 | Supermarket 22 |
| 77 | Chicken luncheon meat | Supplier 3 | Supermarket 22 |
| 78 | Turkey-Chicken Luncheon Meat | Supplier 3 | Supermarket 22 |
| 79 | Beef luncheon meat | Supplier 4 | Supermarket 22 |
| 80 | Chicken luncheon meat | Supplier 4 | Supermarket 22 |
| 81 | Turkey-Chicken Luncheon Meat | Supplier 4 | Supermarket 22 |
| 82 | Beef luncheon meat | Supplier 5 | Supermarket 22 |
| 83 | Chicken luncheon meat | Supplier 5 | Supermarket 22 |
| 84 | Beef luncheon meat | Supplier 6 | Supermarket 22 |
| 85 | Chicken luncheon meat | Supplier 6 | Supermarket 22 |
| 86 | Beef luncheon meat | Supplier 7 | Supermarket 22 |
| 87 | Chicken luncheon meat | Supplier 7 | Supermarket 22 |
| 88 | Beef luncheon meat | Supplier 8 | Supermarket 22 |
| 89 | Chicken luncheon meat | Supplier 8 | Supermarket 22 |
| 90 | Beef luncheon meat | Supplier 9 | Supermarket 23 |
| 91 | Chicken luncheon meat | Supplier 10 | Supermarket 23 |
| 92 | Chicken luncheon meat | Supplier 6 | Supermarket 24 |
| 93 | Chicken luncheon meat | Supplier 4 | Supermarket 24 |
| 94 | Chicken luncheon meat | Supplier 1 | Supermarket 25 |
| 95 | Beef luncheon meat | Supplier 1 | Supermarket 26 |
| 96 | Beef luncheon meat | Supplier 2 | Supermarket 26 |
| 97 | Beef luncheon meat | Supplier 3 | Supermarket 26 |
| 98 | Chicken luncheon meat | Supplier 10 | Supermarket 27 |
| 99 | Chicken luncheon meat | Supplier 4 | Supermarket 27 |
| 100 | Chicken luncheon meat | Supplier 2 | Supermarket 27 |
| 101 | Beef luncheon meat | Supplier 1 | Supermarket 27 |
| 102 | Beef luncheon meat | Supplier 2 | Supermarket 27 |
| 103 | Beef luncheon meat | Supplier 3 | Supermarket 27 |
| 104 | Beef luncheon meat | Supplier 1 | Supermarket 28 |
| 105 | Chicken luncheon meat | Supplier 1 | Supermarket 28 |
| 106 | Turkey-Chicken Luncheon Meat | Supplier 1 | Supermarket 28 |
| 107 | Beef luncheon meat | Supplier 2 | Supermarket 28 |
| 108 | Chicken luncheon meat | Supplier 2 | Supermarket 28 |
| 109 | Turkey-Chicken Luncheon Meat | Supplier 2 | Supermarket 28 |
| 110 | Beef luncheon meat | Supplier 3 | Supermarket 28 |
| 111 | Chicken luncheon meat | Supplier 3 | Supermarket 28 |
| 112 | Turkey-Chicken Luncheon Meat | Supplier 3 | Supermarket 28 |
| 113 | Beef luncheon meat | Supplier 4 | Supermarket 28 |
| 114 | Chicken luncheon meat | Supplier 4 | Supermarket 28 |
| 115 | Turkey-Chicken Luncheon Meat | Supplier 4 | Supermarket 28 |
| 116 | Beef luncheon meat | Supplier 5 | Supermarket 28 |
| 117 | Chicken luncheon meat | Supplier 5 | Supermarket 28 |
| 118 | Beef luncheon meat | Supplier 6 | Supermarket 28 |
| 119 | Chicken luncheon meat | Supplier 6 | Supermarket 28 |
| 120 | Beef luncheon meat | Supplier 7 | Supermarket 28 |
| 121 | Chicken luncheon meat | Supplier 7 | Supermarket 28 |
| 122 | Beef luncheon meat | Supplier 8 | Supermarket 28 |
| 123 | Chicken luncheon meat | Supplier 8 | Supermarket 28 |
| 124 | Beef luncheon meat | Supplier 1 | Supermarket 29 |
| 125 | Chicken luncheon meat | Supplier 1 | Supermarket 29 |
| 126 | Beef luncheon meat | Supplier 2 | Supermarket 30 |
| 127 | Chicken luncheon meat | Supplier 2 | Supermarket 30 |
| 128 | Beef luncheon meat | Supplier 3 | Supermarket 31 |
| 129 | Chicken luncheon meat | Supplier 3 | Supermarket 31 |
| 130 | Beef luncheon meat | Supplier 2 | Supermarket 32 |
| 131 | Chicken luncheon meat | Supplier 2 | Supermarket 32 |
| 132 | Beef luncheon meat | Supplier 1 | Supermarket 33 |
| 133 | Chicken luncheon meat | Supplier 1 | Supermarket 33 |
| 134 | Beef luncheon meat | Supplier 2 | Supermarket 34 |
| 135 | Chicken luncheon meat | Supplier 2 | Supermarket 34 |
| 136 | Beef luncheon meat | Supplier 3 | Supermarket 35 |
| 137 | Chicken luncheon meat | Supplier 3 | Supermarket 35 |
| 138 | Beef luncheon meat | Supplier 1 | Supermarket 36 |
| 139 | Chicken luncheon meat | Supplier 1 | Supermarket 36 |
| 140 | Beef luncheon meat | Supplier 3 | Supermarket 36 |
| 141 | Chicken luncheon meat | Supplier 3 | Supermarket 36 |
| 142 | Beef luncheon meat | Supplier 2 | Supermarket 37 |
| 143 | Chicken luncheon meat | Supplier 2 | Supermarket 37 |
| 144 | Beef luncheon meat | Supplier 3 | Supermarket 37 |
| 145 | Chicken luncheon meat | Supplier 3 | Supermarket 37 |
| 146 | Beef luncheon meat | Supplier 6 | Supermarket 38 |
| 147 | Chicken luncheon meat | Supplier 6 | Supermarket 38 |
| 148 | Beef luncheon meat | Supplier 8 | Supermarket 39 |
| 149 | Chicken luncheon meat | Supplier 8 | Supermarket 39 |
| 150 | Beef luncheon meat | Supplier 7 | Supermarket 40 |
| 151 | Chicken luncheon meat | Supplier 7 | Supermarket 40 |
| 152 | Beef luncheon meat | Supplier 5 | Supermarket 41 |
| 153 | Chicken luncheon meat | Supplier 5 | Supermarket 41 |
| 154 | Beef luncheon meat | Supplier 1 | Supermarket 42 |
| 155 | Beef luncheon meat | Supplier 2 | Supermarket 42 |
| 156 | Beef luncheon meat | Supplier 3 | Supermarket 42 |
| 157 | Beef luncheon meat | Supplier 4 | Supermarket 42 |
| 158 | Beef luncheon meat | Supplier 1 | Supermarket 43 |
| 159 | Beef luncheon meat | Supplier 6 | Supermarket 43 |
| 160 | Beef luncheon meat | Supplier 7 | Supermarket 43 |
| 161 | Chicken luncheon meat | Supplier 7 | Supermarket 43 |
| 162 | Beef luncheon meat | Supplier 1 | Supermarket 44 |
| 163 | Beef luncheon meat | Supplier 2 | Supermarket 44 |
| 164 | Beef luncheon meat | Supplier 3 | Supermarket 44 |
| 165 | Beef luncheon meat | Supplier 4 | Supermarket 44 |
| 166 | Beef luncheon meat | Supplier 5 | Supermarket 44 |
| 167 | Beef luncheon meat | Supplier 6 | Supermarket 44 |
| 168 | Beef luncheon meat | Supplier 7 | Supermarket 44 |
| 169 | Chicken luncheon meat | Supplier 1 | Supermarket 44 |
| 170 | Turkey-Chicken Luncheon Meat | Supplier 1 | Supermarket 44 |
| 171 | Beef luncheon meat | Supplier 2 | Supermarket 45 |
| 172 | Beef luncheon meat | Supplier 4 | Supermarket 45 |
| 173 | Beef luncheon meat | Supplier 6 | Supermarket 45 |
| 174 | Beef luncheon meat | Supplier 7 | Supermarket 45 |
| 175 | Beef luncheon meat | Supplier 8 | Supermarket 45 |
| 176 | Beef luncheon meat | Supplier 1 | Supermarket 46 |
| 177 | Beef luncheon meat | Supplier 2 | Supermarket 46 |
| 178 | Beef luncheon meat | Supplier 3 | Supermarket 46 |
| 179 | Beef luncheon meat | Supplier 4 | Supermarket 46 |
| 180 | Beef luncheon meat | Supplier 5 | Supermarket 46 |
| 181 | Beef luncheon meat | Supplier 7 | Supermarket 46 |
| 182 | Chicken luncheon meat | Supplier 2 | Supermarket 47 |
| 183 | Beef luncheon meat | Supplier 1 | Supermarket 48 |
| 184 | Chicken luncheon meat | Supplier 1 | Supermarket 48 |
| 185 | Turkey-Chicken Luncheon Meat | Supplier 1 | Supermarket 48 |
| 186 | Beef luncheon meat | Supplier 2 | Supermarket 48 |
| 187 | Chicken luncheon meat | Supplier 2 | Supermarket 48 |
| 188 | Turkey-Chicken Luncheon Meat | Supplier 2 | Supermarket 48 |
| 189 | Beef luncheon meat | Supplier 3 | Supermarket 48 |
| 190 | Chicken luncheon meat | Supplier 3 | Supermarket 48 |
| 191 | Turkey-Chicken Luncheon Meat | Supplier 3 | Supermarket 48 |
| 192 | Beef luncheon meat | Supplier 4 | Supermarket 48 |
| 193 | Chicken luncheon meat | Supplier 4 | Supermarket 48 |
| 194 | Beef luncheon meat | Supplier 5 | Supermarket 48 |
| 195 | Chicken luncheon meat | Supplier 5 | Supermarket 48 |
| 196 | Beef luncheon meat | Supplier 6 | Supermarket 48 |
| 197 | Chicken luncheon meat | Supplier 6 | Supermarket 48 |
| 198 | Beef luncheon meat | Supplier 7 | Supermarket 48 |
| 199 | Chicken luncheon meat | Supplier 7 | Supermarket 48 |
| 200 | Beef luncheon meat | Supplier 8 | Supermarket 48 |
| 201 | Chicken luncheon meat | Supplier 8 | Supermarket 48 |
| 202 | Beef luncheon meat | Supplier 1 | Supermarket 49 |
| 203 | Chicken luncheon meat | Supplier 1 | Supermarket 49 |
| 204 | Turkey-Chicken Luncheon Meat | Supplier 1 | Supermarket 49 |
| 205 | Beef luncheon meat | Supplier 2 | Supermarket 49 |
| 206 | Chicken luncheon meat | Supplier 2 | Supermarket 49 |
| 207 | Turkey-Chicken Luncheon Meat | Supplier 2 | Supermarket 49 |
| 208 | Beef luncheon meat | Supplier 3 | Supermarket 49 |
| 209 | Chicken luncheon meat | Supplier 3 | Supermarket 49 |
| 210 | Turkey-Chicken Luncheon Meat | Supplier 3 | Supermarket 49 |
| 211 | Beef luncheon meat | Supplier 4 | Supermarket 49 |
| 212 | Chicken luncheon meat | Supplier 4 | Supermarket 49 |
| 213 | Beef luncheon meat | Supplier 5 | Supermarket 49 |
| 214 | Chicken luncheon meat | Supplier 5 | Supermarket 49 |
| 215 | Beef luncheon meat | Supplier 6 | Supermarket 49 |
| 216 | Chicken luncheon meat | Supplier 6 | Supermarket 49 |
| 217 | Beef luncheon meat | Supplier 7 | Supermarket 49 |
| 218 | Chicken luncheon meat | Supplier 7 | Supermarket 49 |
| 219 | Beef luncheon meat | Supplier 8 | Supermarket 49 |
| 220 | Chicken luncheon meat | Supplier 8 | Supermarket 49 |
| 221 | Beef luncheon meat | Supplier 1 | Supermarket 50 |
| 222 | Beef luncheon meat | Supplier 2 | Supermarket 50 |
| 223 | Beef luncheon meat | Supplier 1 | Supermarket 51 |
| 224 | Beef luncheon meat | Supplier 1 | Supermarket 52 |
| 225 | Beef luncheon meat | Supplier 2 | Supermarket 52 |
| 226 | Beef luncheon meat | Supplier 3 | Supermarket 52 |
| 227 | Beef luncheon meat | Supplier 1 | Supermarket 53 |
| 228 | Beef luncheon meat | Supplier 1 | Supermarket 54 |
| 229 | Chicken luncheon meat | Supplier 1 | Supermarket 54 |
| 230 | Chicken luncheon meat | Supplier 2 | Supermarket 54 |
| 231 | Chicken luncheon meat | Supplier 4 | Supermarket 54 |
| 232 | Beef luncheon meat | Supplier 1 | Supermarket 55 |
| 233 | Beef luncheon meat | Supplier 2 | Supermarket 55 |
| 234 | Beef luncheon meat | Supplier 1 | Supermarket 56 |
| 235 | Beef luncheon meat | Supplier 1 | Supermarket 56 |
| 236 | Beef luncheon meat | Supplier 2 | Supermarket 56 |
| 237 | Beef luncheon meat | Supplier 3 | Supermarket 56 |
| 238 | Beef luncheon meat | Supplier 6 | Supermarket 57 |
| 239 | Beef luncheon meat | Supplier 7 | Supermarket 58 |
| 240 | Beef luncheon meat | Supplier 9 | Supermarket 59 |
| 241 | Beef luncheon meat | Supplier 4 | Supermarket 60 |
| 242 | Chicken luncheon meat | Supplier 4 | Supermarket 60 |
| 243 | Beef luncheon meat | Supplier 1 | Supermarket 61 |
| 244 | Chicken luncheon meat | Supplier 1 | Supermarket 61 |
| 245 | Turkey-Chicken Luncheon Meat | Supplier 1 | Supermarket 61 |
| 246 | Beef luncheon meat | Supplier 2 | Supermarket 61 |
| 247 | Chicken luncheon meat | Supplier 2 | Supermarket 61 |
| 248 | Turkey-Chicken Luncheon Meat | Supplier 2 | Supermarket 61 |
| 249 | Beef luncheon meat | Supplier 3 | Supermarket 61 |
| 250 | Chicken luncheon meat | Supplier 3 | Supermarket 61 |
| 251 | Turkey-Chicken Luncheon Meat | Supplier 3 | Supermarket 61 |
| 252 | Beef luncheon meat | Supplier 4 | Supermarket 61 |
| 253 | Chicken luncheon meat | Supplier 4 | Supermarket 61 |
| 254 | Beef luncheon meat | Supplier 5 | Supermarket 61 |
| 255 | Chicken luncheon meat | Supplier 5 | Supermarket 61 |
| 256 | Beef luncheon meat | Supplier 6 | Supermarket 61 |
| 257 | Chicken luncheon meat | Supplier 6 | Supermarket 61 |
| 258 | Beef luncheon meat | Supplier 7 | Supermarket 61 |
| 259 | Chicken luncheon meat | Supplier 7 | Supermarket 61 |
| 260 | Beef luncheon meat | Supplier 8 | Supermarket 61 |
| 261 | Chicken luncheon meat | Supplier 8 | Supermarket 61 |
| 262 | Beef luncheon meat | Supplier 1 | Supermarket 62 |
| 263 | Chicken luncheon meat | Supplier 1 | Supermarket 62 |
| 264 | Turkey-Chicken Luncheon Meat | Supplier 1 | Supermarket 62 |
| 265 | Beef luncheon meat | Supplier 2 | Supermarket 62 |
| 266 | Chicken luncheon meat | Supplier 2 | Supermarket 62 |
| 267 | Turkey-Chicken Luncheon Meat | Supplier 2 | Supermarket 62 |
| 268 | Beef luncheon meat | Supplier 3 | Supermarket 62 |
| 269 | Chicken luncheon meat | Supplier 3 | Supermarket 62 |
| 270 | Turkey-Chicken Luncheon Meat | Supplier 3 | Supermarket 62 |
| 271 | Beef luncheon meat | Supplier 4 | Supermarket 62 |
| 272 | Chicken luncheon meat | Supplier 4 | Supermarket 62 |
| 273 | Beef luncheon meat | Supplier 5 | Supermarket 62 |
| 274 | Chicken luncheon meat | Supplier 5 | Supermarket 62 |
| 275 | Beef luncheon meat | Supplier 6 | Supermarket 62 |
| 276 | Chicken luncheon meat | Supplier 6 | Supermarket 62 |
| 277 | Beef luncheon meat | Supplier 7 | Supermarket 62 |
| 278 | Chicken luncheon meat | Supplier 7 | Supermarket 62 |
| 279 | Beef luncheon meat | Supplier 8 | Supermarket 62 |
| 280 | Chicken luncheon meat | Supplier 8 | Supermarket 62 |
| 281 | Beef luncheon meat | Supplier 1 | Supermarket 63 |
| 282 | Beef luncheon meat | Supplier 1 | Supermarket 63 |
| 283 | Beef luncheon meat | Supplier 2 | Supermarket 63 |
| 284 | Beef luncheon meat | Supplier 3 | Supermarket 63 |
| 285 | Beef luncheon meat | Supplier 2 | Supermarket 64 |
| 286 | Beef luncheon meat | Supplier 4 | Supermarket 65 |
| 287 | Beef luncheon meat | Supplier 3 | Supermarket 66 |
| 288 | Beef luncheon meat | Supplier 7 | Supermarket 67 |
| 289 | Chicken luncheon meat | Supplier 7 | Supermarket 67 |
| 290 | Beef luncheon meat | Supplier 1 | Supermarket 68 |
| 291 | Chicken luncheon meat | Supplier 1 | Supermarket 68 |
| 292 | Turkey-Chicken Luncheon Meat | Supplier 1 | Supermarket 68 |
| 293 | Beef luncheon meat | Supplier 2 | Supermarket 68 |
| 294 | Chicken luncheon meat | Supplier 2 | Supermarket 68 |
| 295 | Turkey-Chicken Luncheon Meat | Supplier 2 | Supermarket 68 |
| 296 | Beef luncheon meat | Supplier 3 | Supermarket 68 |
| 297 | Chicken luncheon meat | Supplier 3 | Supermarket 68 |
| 298 | Turkey-Chicken Luncheon Meat | Supplier 3 | Supermarket 68 |
| 299 | Beef luncheon meat | Supplier 4 | Supermarket 68 |
| 300 | Chicken luncheon meat | Supplier 4 | Supermarket 68 |
| 301 | Beef luncheon meat | Supplier 5 | Supermarket 68 |
| 302 | Chicken luncheon meat | Supplier 5 | Supermarket 68 |
| 303 | Beef luncheon meat | Supplier 6 | Supermarket 68 |
| 304 | Chicken luncheon meat | Supplier 6 | Supermarket 68 |
| 305 | Beef luncheon meat | Supplier 7 | Supermarket 68 |
| 306 | Chicken luncheon meat | Supplier 7 | Supermarket 68 |
| 307 | Beef luncheon meat | Supplier 8 | Supermarket 68 |
| 308 | Chicken luncheon meat | Supplier 8 | Supermarket 68 |
| 309 | Beef luncheon meat | Supplier 2 | Supermarket 69 |
| 310 | Chicken luncheon meat | Supplier 2 | Supermarket 69 |
| 311 | Beef luncheon meat | Supplier 1 | Supermarket 70 |
| 312 | Chicken luncheon meat | Supplier 1 | Supermarket 70 |
| 313 | Beef luncheon meat | Supplier 3 | Supermarket 71 |
| 314 | Chicken luncheon meat | Supplier 3 | Supermarket 71 |
| 315 | Beef luncheon meat | Supplier 1 | Supermarket 72 |
| 316 | Beef luncheon meat | Supplier 2 | Supermarket 72 |
| 317 | Beef luncheon meat | Supplier 3 | Supermarket 72 |
| 318 | Beef luncheon meat | Supplier 2 | Supermarket 73 |
| 319 | Beef luncheon meat | Supplier 4 | Supermarket 73 |
| 320 | Beef luncheon meat | Supplier 5 | Supermarket 73 |
| 321 | Beef luncheon meat | Supplier 1 | Supermarket 74 |
| 322 | Chicken luncheon meat | Supplier 1 | Supermarket 74 |
| 323 | Turkey-Chicken Luncheon Meat | Supplier 1 | Supermarket 74 |
| 324 | Beef luncheon meat | Supplier 2 | Supermarket 74 |
| 325 | Chicken luncheon meat | Supplier 2 | Supermarket 74 |
| 326 | Turkey-Chicken Luncheon Meat | Supplier 2 | Supermarket 74 |
| 327 | Beef luncheon meat | Supplier 3 | Supermarket 74 |
| 328 | Chicken luncheon meat | Supplier 3 | Supermarket 74 |
| 329 | Turkey-Chicken Luncheon Meat | Supplier 3 | Supermarket 74 |
| 330 | Beef luncheon meat | Supplier 4 | Supermarket 74 |
| 331 | Chicken luncheon meat | Supplier 4 | Supermarket 74 |
| 332 | Beef luncheon meat | Supplier 5 | Supermarket 74 |
| 333 | Chicken luncheon meat | Supplier 5 | Supermarket 74 |
| 334 | Beef luncheon meat | Supplier 6 | Supermarket 74 |
| 335 | Chicken luncheon meat | Supplier 6 | Supermarket 74 |
| 336 | Beef luncheon meat | Supplier 7 | Supermarket 74 |
| 337 | Chicken luncheon meat | Supplier 7 | Supermarket 74 |
| 338 | Beef luncheon meat | Supplier 8 | Supermarket 74 |
| 339 | Chicken luncheon meat | Supplier 8 | Supermarket 74 |
| 340 | Beef luncheon meat | Supplier 1 | Supermarket 75 |
| 341 | Beef luncheon meat | Supplier 2 | Supermarket 75 |
| 342 | Beef luncheon meat | Supplier 3 | Supermarket 75 |
| 343 | Beef luncheon meat | Supplier 6 | Supermarket 76 |
| 344 | Beef luncheon meat | Supplier 7 | Supermarket 76 |
| 345 | Beef luncheon meat | Supplier 2 | Supermarket 77 |
| 346 | Beef luncheon meat | Supplier 3 | Supermarket 77 |
| 347 | Beef luncheon meat | Supplier 1 | Supermarket 78 |
| 348 | Beef luncheon meat | Supplier 4 | Supermarket 78 |
| 349 | Beef luncheon meat | Supplier 5 | Supermarket 79 |
| 350 | Chicken luncheon meat | Supplier 5 | Supermarket 79 |
