# Supplementary material for: Characterization of β-lactam resistance in K. pneumoniae associated with ready-to-eat processed meat in Egypt
Source: PLoS One. 2020 Sep 3;15(9):e0238747. doi: 10.1371/journal.pone.0238747 (PMC7470258; doi:10.1371/journal.pone.0238747)
Supplement: S2 Table — (DOCX) [file pone.0238747.s002.docx]

**S2 Table: Classification of *K. pneumoniae* according to biochemical reactions**

| **Biochemical test** | **Biotypes** | | | | |
| --- | --- | --- | --- | --- | --- |
|  | **B1** | **B2** | **B3** | **B4** | **B5** |
| **Motility** | **-** | **-** | **-** | **-** | **-** |
| **Indole** | **-** | **-** | **-** | **-** | **-** |
| **Methyle red (MR)** | **-** | **-** | **-** | **-** | **-** |
| **Voges Proskuaer (VP)** | **+** | **+** | **+** | **+** | **+** |
| **Citrate utilization** | **+** | **+** | **-** | **+** | **-** |
| **Urease** | **+** | **+** | **+** | **-** | **+** |
| **H2S** | **-** | **-** | **-** | **-** | **-** |
| **Gelatin liquefaction** | **-** | **-** | **-** | **-** | **-** |
| **ODC** | **-** | **-** | **-** | **-** | **-** |
| **LDC** | **-** | **-** | **+** | **+** | **+** |
| **Arginine dihydrolase** | **-** | **-** | **-** | **-** | **-** |
| **ONPG** | **+** | **+** | **+** | **+** | **+** |
| **Sugar fermentation**  **Lactose** | **+** | **+** | **+** | **+** | **+** |
| **Glucose** | **+** | **+** | **+** | **+** | **+** |
| **Mannitol** | **+** | **+** | **+** | **+** | **+** |
| **Inositol** | **+** | **+** | **+** | **+** | **-** |
| **Sorbitol** | **+** | **+** | **+** | **+** | **+** |
| **Rhamnose** | **+** | **-** | **+** | **+** | **+** |
| **Arabinose** | + | + | + | + | + |
